# Use of High-density Mapping to Ablate a Wide Accessory Pathway and Mahaim Fiber in a Patient with Ebstein’s Anomaly

**DOI:** 10.19102/icrm.2024.15041

**Published:** 2024-04-15

**Authors:** Eric Rosenthal, Ka-Chun Un, Julian Bostock

**Affiliations:** 1 Department of Paediatric Cardiology, Evelina London Children’s Hospital, Guy’s and St Thomas’ NHS Foundation Trust, London, UK

**Keywords:** Catheter ablation, Ebstein’s anomaly, high-density mapping

## Abstract

Ablation of accessory pathways in patients with Ebstein’s anomaly can be challenging. Despite increasing experience and advances in mapping technology, success is limited and recurrence rates can be high. To date, high-density electroanatomic mapping has not been studied in this anatomical substrate. We present a pediatric case of Ebstein’s anomaly in which high-density mapping in Ebstein’s anomaly was a useful additional tool to improve the outcome of catheter ablation.

## Introduction

Ablation of accessory pathways in patients with Ebstein’s anomaly can be challenging due to the displacement of the anatomical annulus from the electrical annulus; low-amplitude signals over the atrialized portion of the right ventricle; and multiple accessory pathways, which can be broad. Despite increasing experience and advances in mapping technology, there is a lower success rate and a higher recurrence rate.^1–3^ High-density electroanatomic mapping has not been studied in this anatomical substrate. Additionally, in some patients, a Mahaim fiber coexists, adding to the complexity.^4,5^

## Case presentation

**Figure 1:**
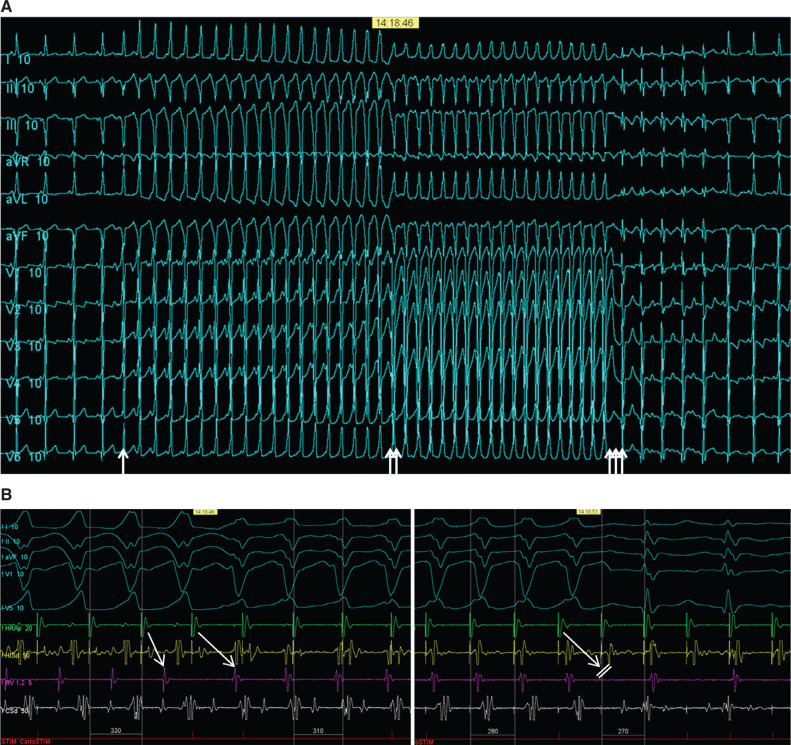
Effect of incremental atrial pacing. **A:** Baseline pre-excitation is accentuated (arrow), following which there is a change to a more typical left bundle branch block pattern (double arrow) and then onset of 2:1 atrioventricular (AV) block with a narrow QRS and incomplete right bundle branch block pattern (triple arrow). **B:** Electrograms from **Figure 1A**. Left panel shows maximum pre-excitation for four beats followed by a change in pattern to a narrower left bundle branch block pattern preceded by a lengthening in the AV interval (first and second arrows; corresponding to the double arrow in **Figure 1A**). The right panel shows the onset of 2:1 AV block with a narrow QRS and an incomplete right bundle branch block pattern (third arrow; corresponding to triple arrow in **Figure 1A**).

**Figure 2:**
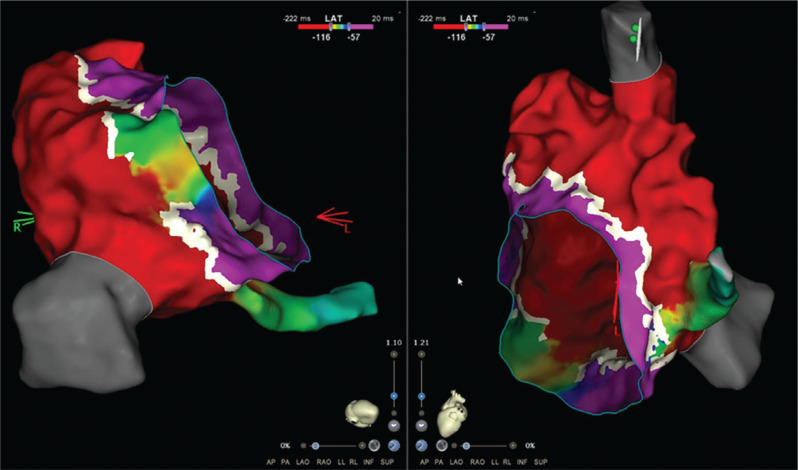
Open-window map during atrial pacing. The white line represents the atrioventricular junction with the atrium in red and ventricle in purple. There is a broad area of conduction across the right posterior (inferior) atrioventricular junction in green/yellow.

**Figure 3:**
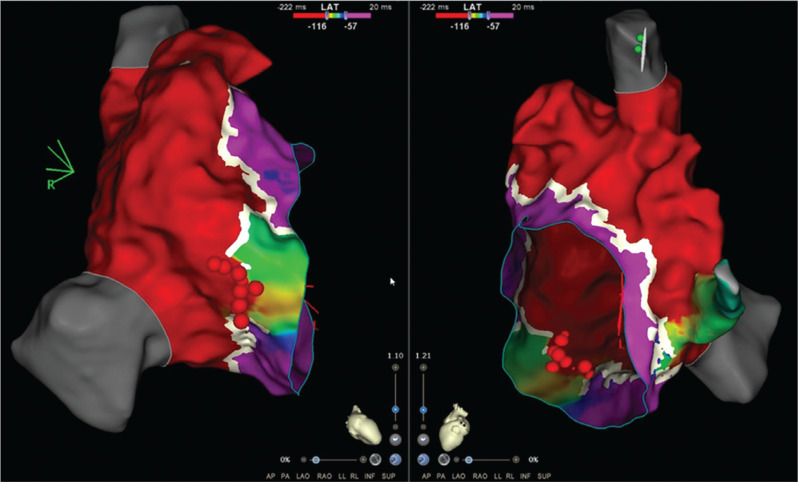
Linear ablation lesion set. Modified right and left anterior oblique views showing ablation points in red “plugging” the gap in the open-window map, resulting in a loss of pre-excitation.

**Figure 4:**
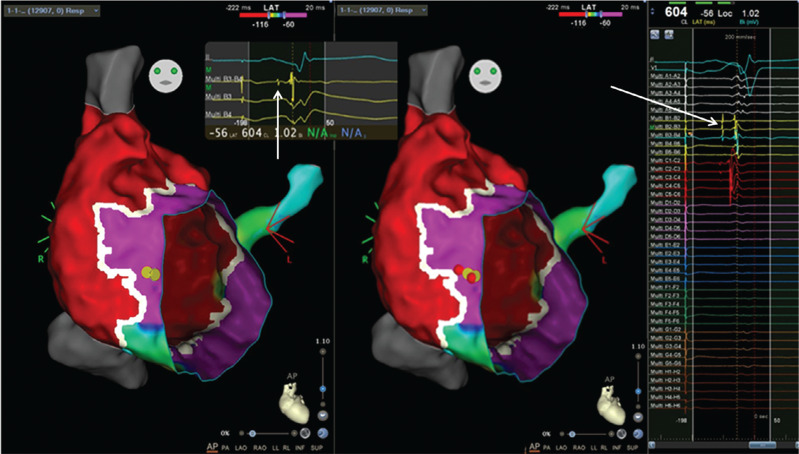
Ablation of Mahaim fiber. The panel on the left shows yellow dots marking the Mahaim potential (white arrow) seen in yellow electrograms on the far-right panel showing the 40 electrograms obtained simultaneously with the Octaray catheter (signals magnified inset). Panel in the center shows the red ablation lesions guided by the mapping with abolition of Mahaim conduction.

An 11-year-old boy with Ebstein’s anomaly and moderate tricuspid valve displacement but only mild tricuspid regurgitation with overt pre-excitation developed supraventricular tachycardia and underwent a fluoroscopy-free electrophysiology study. During incremental atrial pacing, the accessory pathway became more overt, then changed to a pure left bundle branch block (LBBB) pattern with prolongation of the atrioventricular (AV) time before 2:1 AV block ensued with conduction across the AV node, revealing an underlying incomplete right bundle branch block (RBBB) pattern **(Figures 1A and 1B)**. Atrial extrastimulus pacing induced runs of antidromic atrioventricular re-entrant tachycardia (AVRT) with an LBBB pattern and a long P–R interval. High-density mapping of the AV ring was performed during atrial pacing with maximum pre-excitation using an Octaray multipolar catheter with 2-mm spacing between electrodes on eight splines (Biosense Webster, Diamond Bar, CA, USA). Mapping was aided by a Vizigo sheath (Biosense Webster) in the difficult-to-reach areas. Open-window mapping (14,000 points) revealed a broad area of AV conduction on the posterior tricuspid annulus with continuous AV signals **(Figure 2)**. Several ablations (Cool flow Navistar; Biosense Webster) in this area had a transient effect on accessory pathway conduction, but, after a linear row of lesions was delivered to the gap in the open-window map, there was no further conduction over the accessory pathway **(Figure 3)**. Incremental atrial pacing now elicited an LBBB pattern that was not pre-excited, and antidromic AVRT was still inducible with the same pattern, suggestive of a Mahim fiber. A review of the high-density mapping electrograms revealed a “His-like” deflection at about 9 o’clock on the tricuspid annulus. The ablation catheter was unable to record a similar signal, even with amplification. Catheter pressure at the sites indicated by the electrograms on the open-window map resulted in the cessation of Mahaim conduction. Ablation was performed with no recurrence of Mahaim fiber conduction **(Figure 4)**. The final ECG showed no pre-excitation with an incomplete RBBB pattern with decremental AV and ventriculoatrial conduction and non-inducibility of AVRT.

The patient’s parent provided written informed consent for case publication.

## Discussion

Open-window mapping has recently been found to be useful in aiding ablation of conventional accessory AV pathways.^6–8^ In Ebstein’s anomaly, the AV annulus can be poorly defined due to the adherence of the tricuspid valve tissue to the ventricular myocardium, creating a zone of atrialized ventricular myocardium. This zone often has low-voltage signals that can be difficult to interpret and makes definition of the true electrical AV annulus difficult. The adherence of the tricuspid valve to the ventricular myocardium is also associated with multiple and broader accessory AV pathways. These features, together with poor catheter stability, can make ablation of accessory pathways in Ebstein’s anomaly challenging, with a lower success rate and a higher recurrence rate.^1–3^

High-density mapping with the open-window algorithm was initially beneficial both in identifying the electrical annulus and also demonstrating a broad area of accessory pathway conduction. Point ablations guided by signals were ineffective until a row of lesions was placed to “plug” the gap in the open-window map.

Mahaim fibers are also a feature of Ebstein’s syndrome, and mapping of these can be challenging, especially when the annulus is ill-defined.^4,5^ The Mahaim fiber signal on the normal AV annulus is not uncommonly indistinct when mapping with large surface area catheters, and these robust catheters may also block conduction inadvertently, preventing ablation. The high-density electrograms recorded with the delicate narrow-spaced electrodes of the Octaray catheter were able to identify the Mahaim potential clearly when the larger surface area electrodes of the ablation catheter were unable to detect it. This guided the ablation catheter to the site and allowed an effective ablation. High-density mapping has been used once before to demonstrate the potentials of a purely retrograde decremental accessory pathway in a concealed Mahaim-type structure on the right lateral AV groove in a 22-year-old man with hypertrophic cardiomyopathy and a normal tricuspid valve.^9^

## Conclusions

High-density mapping in Ebstein’s anomaly may prove to be a useful additional tool to improve the outcome of catheter ablation in this anatomical substrate.
